# Supplementary material for: Hard to Reach and Hidden: Improving the Identification of Young Dementia Carers
Source: Int J Environ Res Public Health. 2023 Nov 23;20(23):7103. doi: 10.3390/ijerph20237103 (PMC10871087; doi:10.3390/ijerph20237103)
Supplement: Supplementary file 1 [file ijerph-20-07103-s001.zip › Supplementary File S1_Masterson-Algar et al.pdf]

Exploring recruitment (young dementia carers)

Example interview schedule

Introduction:

Many thanks for taking the time to help with this interview.

This interview is being carried out to gather information about how we can improve the visibility of young dementia carers. If at any time you want to stop, have a break, or if you don't want to answer a question, please let me know. Please be assured that everything you say will remain confidential. Extracts of what you say may be included in publications or reports, but your name or any other confidential details will not be linked to it.

Can I ask you to confirm that you are happy to continue, and for the conversation to be recorded? If you are not, I can make notes instead.

1. **Please can you tell me about your experience of working with YC? How long have you worked with YCs?**
2. **What is your experience of identifying YCs? Is there a particular group that is more vulnerable? What do you think is the impact of demographics (level of relative deprivation)?**
3. **Would you say that kids that are already 'flagged up' will then be more likely to be identified as YC and supported?**
4. **Please tell me about your experience of dementia and young dementia carers.**

    - Has your family been impacted by dementia? In what way?
    - What is your link to YDC?
    - How long have you been working in the area of dementia? What is your role?
    - Have you worked with YDC? Can you remember when you first had contact with this particular group?
    - Do you regularly have contact with families affected by dementia and young onset dementia?
    - In your work would you know what to do if a child came to you and said that their parent/grandparent has dementia?

5. **Young dementia carers – what defines this group?**

    - From your experience what is the most effective way to identify these children?
    - **When diagnosis came, did any health professional ask anything about the young family members?**
    - **The issue around diagnosis and disclosure -** How did you tell your kids about the diagnosis of their family member? When was the right time? Did

you want them to know? Do you think families want their kids to know? Is it possible to hide it for some time?

- When did you think 'my children need support'?
- From your experience what are the common characteristics of these children?
- Why do you think they need support? What type of support?
- The nature of dementia may play a role in these kids remaining invisible, would you agree? Why? What is the biggest barrier to identification?
- Do you think the progression of the illness plays a role? Why? When is the best time to start supporting these children?

6. **What do you think about the role of schools and YCs?**

- What can they do? How do they identify YCs? Why do the details around 'illness' don't seem to be a factor? **(e.g., questionnaires don't ask about the condition or illness of family member – only the MACA and PANOC)**
- Do schools actively ask parents about their kids (so they could disclose the family situation even if the child doesn't want to)? **Do parents get a survey asking about it?**
- Do you have personal experience of this? Can you explain?
- What would you have changed?

7. **What do you think about the role of charities and similar organizations?**

- What can they do?
- Do you have personal experience of this? Can you explain?
- What would you have changed?

8. **What is the support for YDC currently available?**

- What do you think about the resources that are out there? Is there something missing? What?
- Is it tailored to them? Do they access it?

9. **Identifying (recruiting) these YDC**

- What methods would you use to identify YDCs?
- How and what avenues would you use to identify YDCs?

10. **The role of Social Media**

- Does it help or not? In what way? Practicalities (e.g., proof of age)
- Is it the right avenue to identify YDCs? Why?

To end: I have asked you all the questions I wanted to ask you, is there anything you would like to say or any other issue I haven't mentioned that you would like to discuss?

Thank you very much for your help.
