# Supplementary material for: Hard to Reach and Hidden: Improving the Identification of Young Dementia Carers
Source: Int J Environ Res Public Health. 2023 Nov 23;20(23):7103. doi: 10.3390/ijerph20237103 (PMC10871087; doi:10.3390/ijerph20237103)

Aim

1. Give you the chance to 'check' preliminary themes

2. Discuss pathways to increase the visibility (and identify) young dementia carers

3. Think about 'next steps'

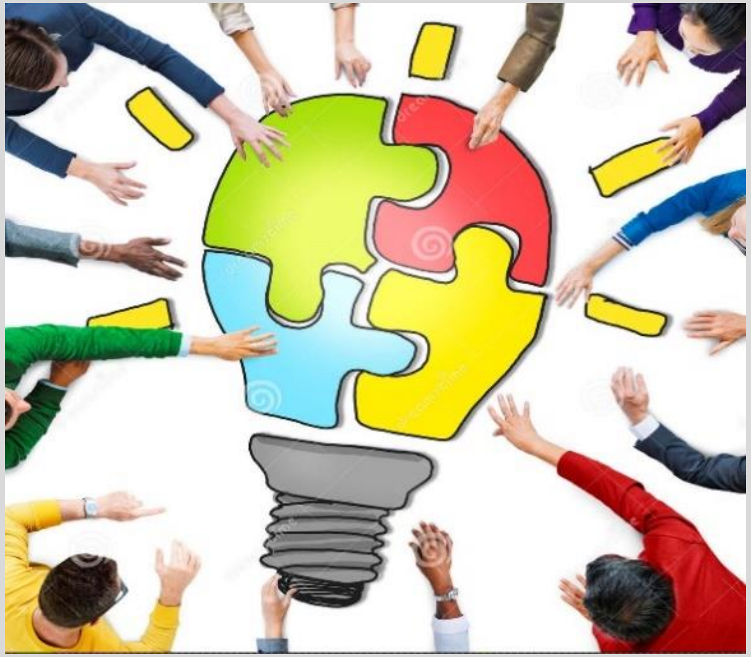

Did I get it right?

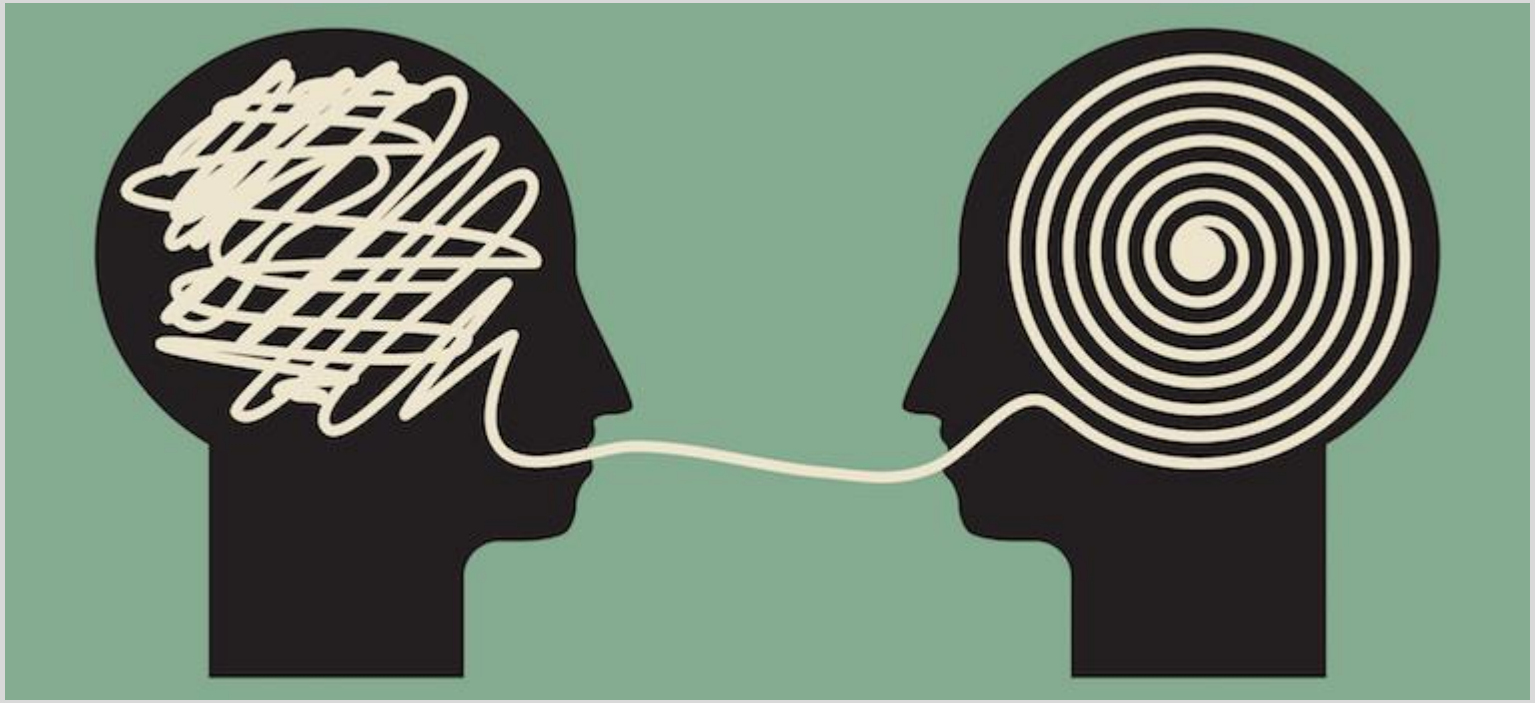

Theme 1:
It needs to be a 'whole family approach'

Theme 2:
Nothing fancy – A chat is sometimes all it's needed

Theme 3:
No voice / No presence

Theme 4:
Postcode/School lottery

Theme 5:
Need to 'link' with adult campaigns/initiatives
(Unite forces)

Theme 6:
It feels like nothing changes

Theme 7:
I am not a carer. This is my 'normal'

Identification/Communication

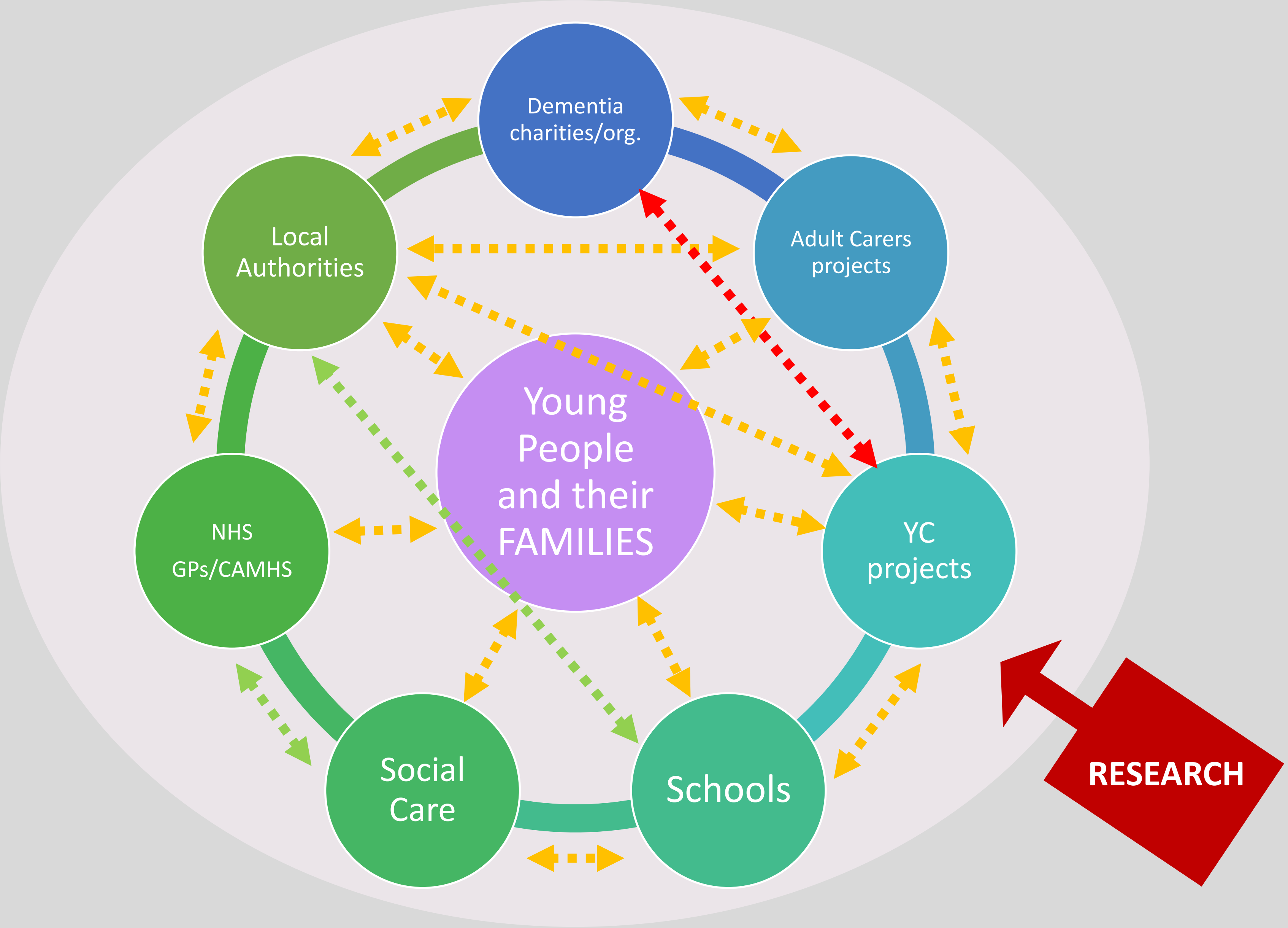

Identification/Communication

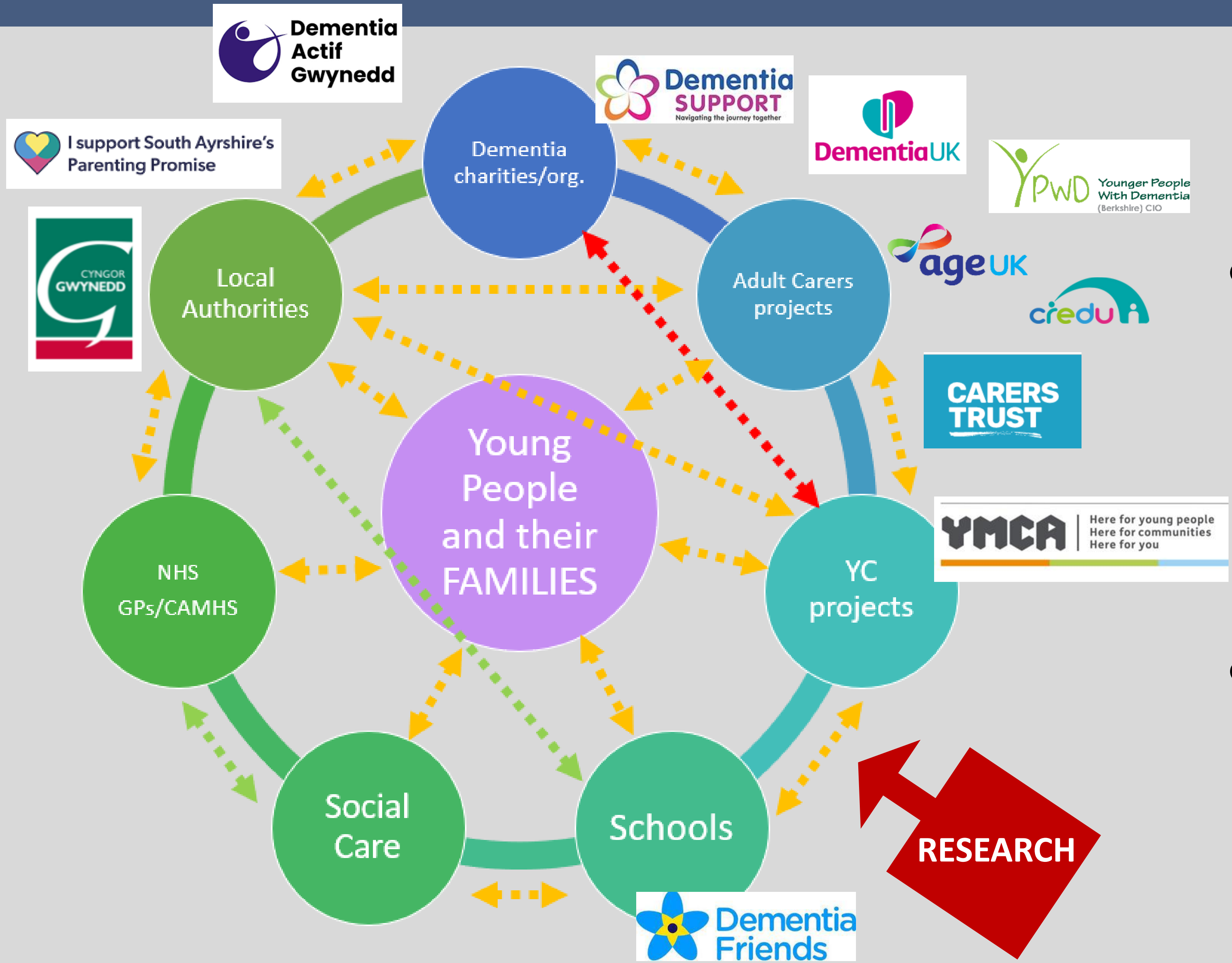

- How can these communication pathways improve?
- What can you/your organization do?

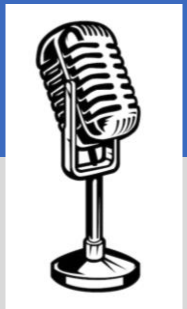

Increasing VISIBILITY

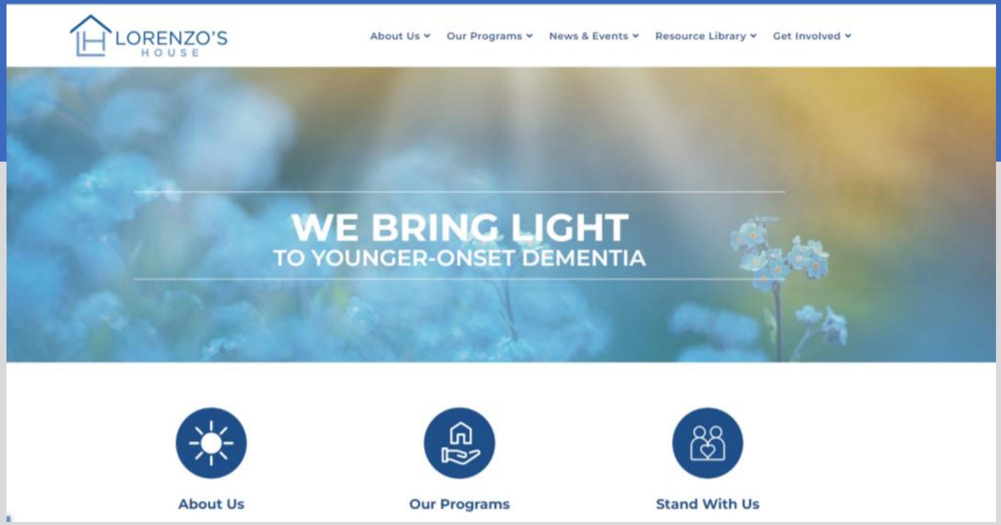

- Online Network (e.g. similar model as Lorenzo's House) – including peer support
- Creation of small clips/resources that can be embedded in current 'information and awareness' sessions
- **Podcast** led by young people

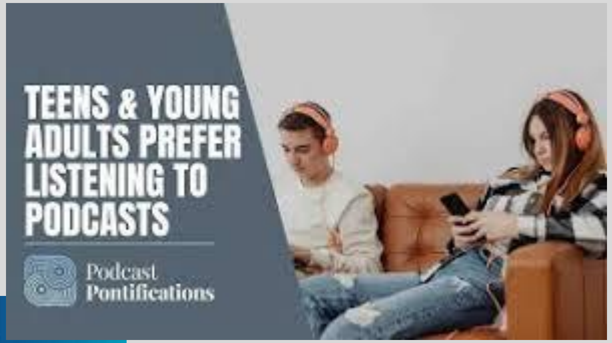

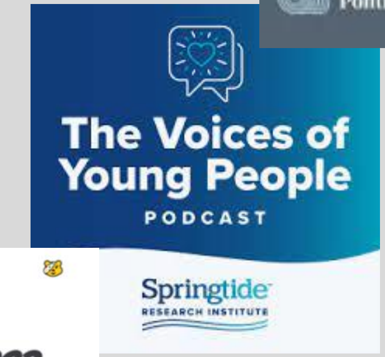

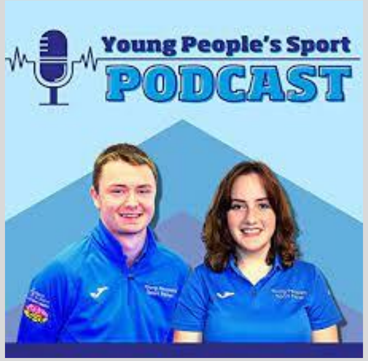

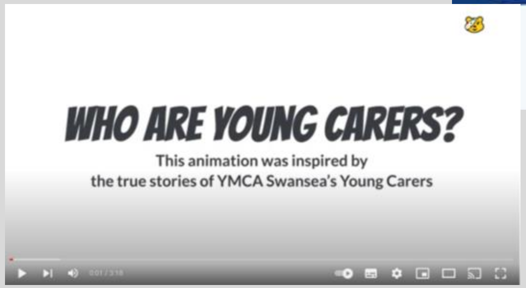

Tailored support – at the right time

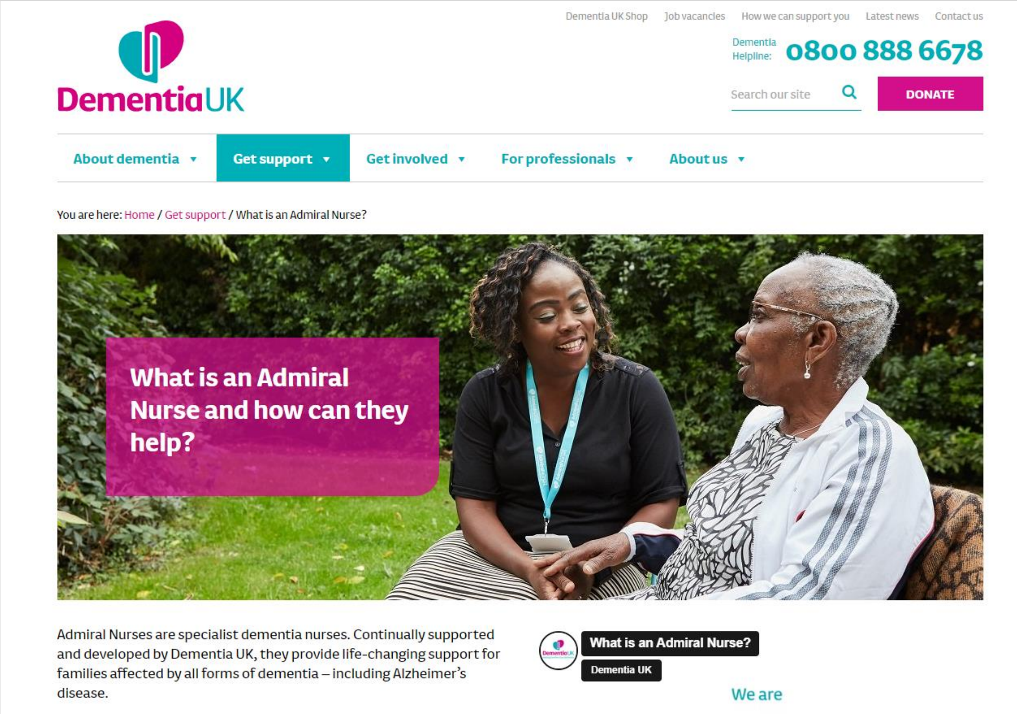

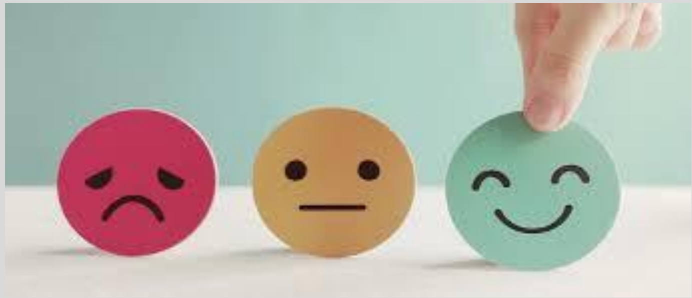

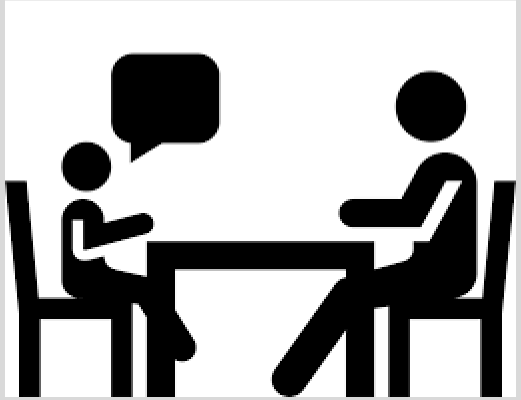

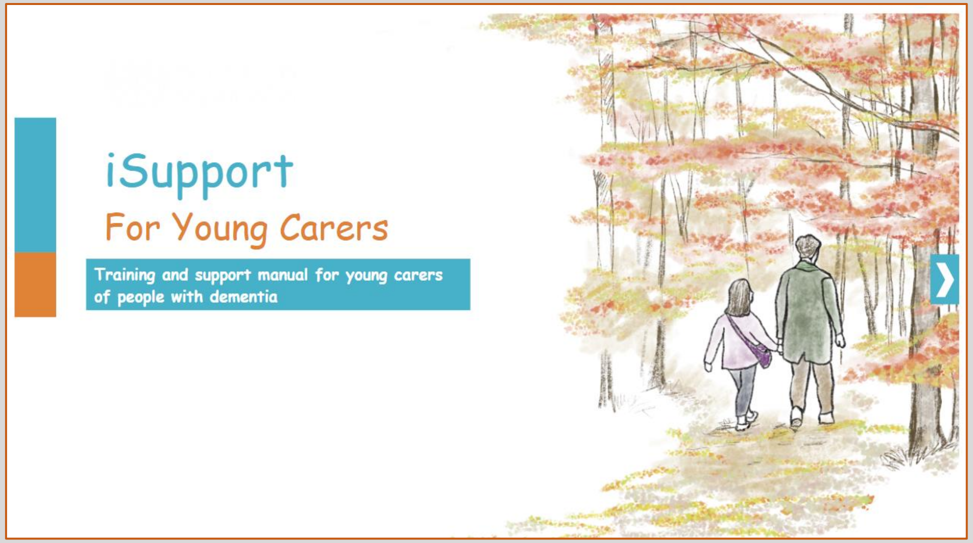

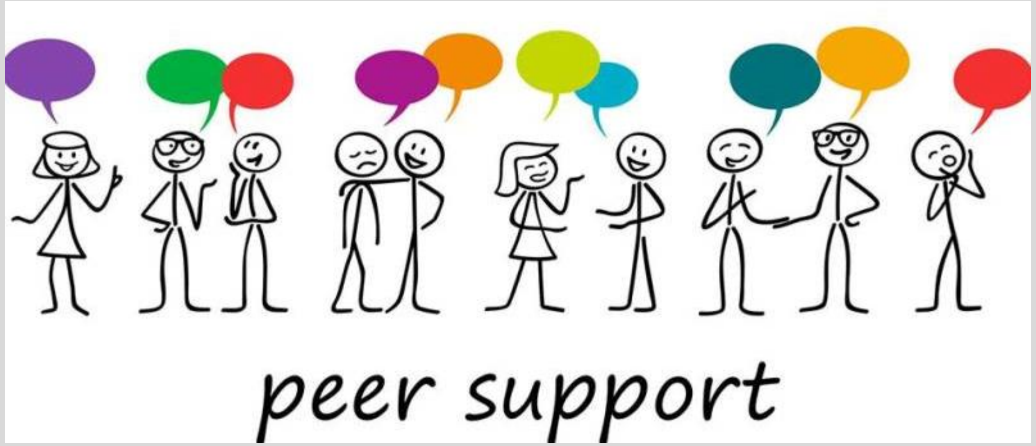

What next?

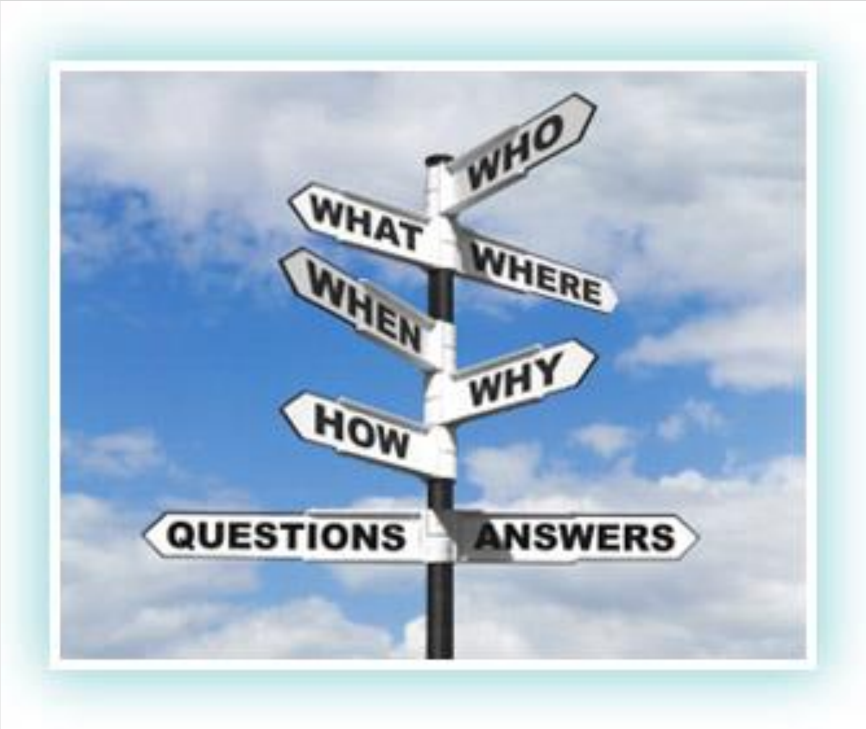

Supplement: Supplementary file 1 [file ijerph-20-07103-s001.zip › Supplementary File S2_Masterson-Algar et al. 2023.pdf]
